# Circulating C3 is necessary and sufficient for induction of autoantibody-mediated arthritis in a mouse model

**DOI:** 10.1002/art.22859

**Published:** 2007-09

**Authors:** Paul A Monach, Admar Verschoor, Jonathan P Jacobs, Michael C Carroll, Amy J Wagers, Christophe Benoist, Diane Mathis

**Affiliations:** 1 Brigham and Women's Hospital and Harvard Medical SchoolBoston, Massachusetts; 2 Harvard Medical SchoolBoston, Massachusetts; 3 Harvard Medical SchoolBoston, Massachusetts

## Abstract

**Objective:**

For the inflammation characteristic of rheumatoid arthritis, the relative contribution of mediators produced locally in the synovium versus those circulating systemically is unknown. Complement factor C3 is made in rheumatoid synovium and has been proposed to be a crucial driver of inflammation. The aim of this study was to test, in a mouse model of rheumatoid arthritis, whether C3 synthesized within the synovium is important in promoting inflammation.

**Methods:**

Radiation bone marrow chimeras between normal and C3^−/−^ mice were constructed in order to generate animals that expressed or lacked expression of C3 only in hematopoietic cells. Parabiotic mice were made by surgically linking C3^−/−^ mice to irradiated wild-type mice to obtain animals having C3 only in the circulation. Arthritis was induced by injection of serum from arthritic K/BxN mice.

**Results:**

In bone marrow chimeras, synthesis of C3 by radioresistant cells was necessary and sufficient to confer susceptibility to serum-transferred arthritis. Parabionts having C3 only in the circulation remained sensitive to arthritis induction, and the cartilage of these arthritic mice contained deposits of C3.

**Conclusion:**

In a mouse model in which the alternative pathway of complement activation is critical to the induction of arthritis by autoantibodies, circulating C3 was necessary and sufficient for arthritis induction.

The complement cascade is essential for the induction of inflammatory arthritis by autoantibodies in at least 2 mouse models (1–3). The role of complement in human rheumatoid arthritis (RA) has been more difficult to assess, but a contribution of this pathway is suggested by several findings. First, complement components are depleted (4,5) and complement degradation products are generated (6,7) in the synovial fluid in RA but not other types of inflammatory arthritis. Second, C3 is deposited on the surface of cartilage and synovium in RA (8,9), as it is in various rodent models (10–12).

The details of complement involvement are particularly clear in the K/BxN mouse serum-transfer model. K/BxN mice uniformly develop severe, symmetric, inflammatory arthritis due to activation of the KRN transgene-encoded T cell receptor by a peptide from the glycolytic enzyme glucose-6-phosphate isomerase (GPI) presented by the class II major histocompatibility complex molecule A^g7^ (13), leading to massive production of anti-GPI antibodies. These antibodies can effectively induce arthritis upon transfer into other mice (14). Because a wide range of natural mutant and gene-disrupted mouse strains can be used as recipients, this serum-transfer model has allowed the delineation of many genes and cell types required downstream of autoantibody production (1,15–18). With regard to the complement cascade, factors B, D (Monach PA: unpublished observations), C3, C5, and the receptor for C5a (C5aR) are required, whereas C1q, C4, C6, and the complement receptors CR1, CR2, and CR3 are not (1,19). Thus, induction of arthritis requires the alternative pathway of complement activation, leading to production of the chemoattractant and activating mediator C5a. Recently, a similar requirement for alternative but not classical pathway elements was found for induction of arthritis by antibodies directed against type II collagen (20). Most studies of complement in RA have not differentiated between activation of the classical and alternative pathways, but one that did so indicated that local activation of the alternative pathway in synovial fluid is particularly characteristic of RA (21).

The details of C3 involvement in inflammatory arthritis are of particular interest, not only because this protein is involved in all of the major pathways of complement activation and subsequent activation of effector mechanisms, but also because both systemic and local synthesis have been well documented. A few years ago, one might have assumed that the obligatory source of C3 and other essential complement components would be the liver. The liver is thought to be the source of the vast majority of circulating C3, and although this protein has a relatively short half-life, its concentration in plasma is the highest of any complement protein, at 1.0–1.4 mg/ml. However, not only has the synthesis of complement proteins by leukocytes now been clearly demonstrated (22–24), but leukocyte-derived C3 was found to be sufficient for the generation of antibody responses to a model antigen (25) and to be both necessary and sufficient for optimal antibody responses to intradermal herpes simplex virus infection in mice (26,27). Production of C3 by the inflamed synovium from patients with RA has also been demonstrated (28), and both hematopoietic and nonhematopoietic cells were implicated as potential sources (29,30), leading to the proposal that local synthesis of C3 might be important in propagating inflammation (30).

Because it is not currently possible to test this hypothesis in human RA, we did so in the K/BxN mouse serum-transfer system by using bone marrow chimeras and parabiotic mice.

## MATERIALS AND METHODS

### Mice

C3^−/−^ mice (31) were maintained locally; C57BL/6 (B6) mice and B6 mice congenic for the CD45.1 isoform were purchased from The Jackson Laboratory (Bar Harbor, ME). Animals were maintained under specific pathogen–free conditions, and all procedures were performed in accordance with Institutional Animal Care and Use Committee–approved protocols ARCM-03204 and ARCM-03912.

### Bone marrow chimeras

Recipient mice were lethally irradiated (6.5 Gy administered twice, 6 hours apart) and reconstituted intravenously with unfractionated bone marrow cells (BMCs) freshly obtained from the femurs of donor mice. Staining for the CD45.1 and CD45.2 isoforms on peripheral blood leukocytes (PBLs) showed that >95% of PBLs were of donor origin. Circulating C3 was measured by enzyme-linked immunosorbent assay (ELISA) and correlated perfectly with the capacity of the recipient to synthesize C3 (26). Chimeric animals were tested for arthritis susceptibility 6–12 weeks after reconstitution.

### Parabiotic mice

Wild-type (WT) mice were lethally irradiated (10 Gy) immediately prior to parabiosis surgery. As described previously (32,33), animals were anesthetized and the skin incised from elbow to knee on one side. The elbows and knees of a pair of mice were sutured together through the musculature, then the skin incision was stapled and sutured such that the animals were joined by the dorsal and ventral skin. Parabionts were given parenteral analgesia for 2 days and trimethoprim/sulfamethoxazole in the drinking water for 2 weeks. In a pilot experiment using pairs of WT mice mismatched for CD45 isoforms, leukocytes from the nonirradiated partner were readily detectable in the irradiated partner by 7 days after surgery and comprised >90% of the circulating leukocytes. Parabionts were tested for susceptibility to arthritis 5 weeks after surgery.

### Arthritis induction

K/BxN mice were bred as described previously (34). Serum was collected at 7 weeks of age and stored at −20°C. Serum was injected intraperitoneally by our standard protocol (14), as follows: 0.15 ml on day 0 and again on day 2. The 4 paws were assessed by the clinical score (0–3-point scale for each paw, where 0 = no swelling, 1 = either swelling confined to 1 or 2 digits or mild swelling of the larger structures, 3 = severe arthritis involving the wrist or ankle but extending along the dorsum of the paw to the bases of the digits, and 2 = intermediate severity [score between 1 and 3]). In addition, thickening of the right ankle (or of the unconstrained ankle in parabiotic mice) was measured using a precision caliper (Kafer dial thickness gauge with flat anvils; Long Island Indicator Service, Hauppauge, NY). Disease was routinely evaluated twice per week.

### Histologic assessment and immunofluorescence staining

For frozen sections, the skin over an ankle was removed immediately after the animal was killed, and a piece of tissue ∼10 mm long encompassing the ankle and midfoot joints was immersed in Tissue-Tek OCT medium (Sakura Finetek, Torrance, CA), flash-frozen in dry ice/ethanol, and stored at −80°C until sectioned. Ankles were sectioned, without prior decalcification, using a tape transfer method (Instrumedics, Hackensack, NJ), as described previously (1,16). Sections were stored at −20°C until used, at which time they were fixed for 5 minutes in acetone at 4°C, briefly allowed to dry, rehydrated with phosphate buffered saline (PBS) for 15 minutes, blocked with PBS containing 2% bovine serum albumin (BSA) and 0.1% Tween 20 for 45 minutes, stained with fluorescein-labeled antibody to C3 (F[ab′]_2_ goat anti-mouse C3; ICN Biochemicals, Irvine, CA), diluted 1:200 in PBS/0.1% Tween 20 for 60 minutes, then washed 3 times with PBS/Tween 20 before placing coverslips using Gel/Mount medium (Biomeda, Foster City, CA).

For paraffin sections, fresh tissue containing the ankle and midfoot joints was fixed in 4% paraformaldehyde at 4°C overnight, decalcified with 3 changes of 0.375*M* EDTA (pH 7.5) at 4°C for 2 weeks, and then processed for paraffin sectioning and hematoxylin and eosin staining by standard techniques.

Microscopy was performed using a Zeiss Axioplan 2 instrument equipped with a Spot RT Slider camera (Diagnostic Instruments, Sterling Heights, MI) and IPLab imaging software (Scanalytics, Billerica, MA).

### Measurement of C3

Circulating C3 was measured by ELISA, as previously described (26).

## RESULTS

### A requirement for C3 synthesis by radioresistant cells

**Figure 1:**
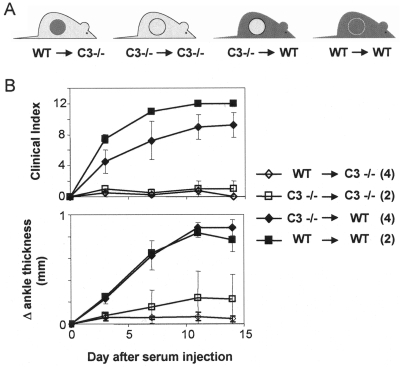
Arthritis susceptibility in chimeric mice. **A,** All combinations of wild-type (WT) or C3^−/−^ donors and recipients were used. Recipients were lethally irradiated, then reconstituted with unfractionated bone marrow cells. Five to 12 weeks after bone marrow reconstitution, mice were injected with 0.15 ml K/BxN mouse serum twice, 2 days apart. **B,** Arthritis was evaluated qualitatively by clinically assessing the severity in all 4 paws and quantitatively by measuring the thickness of the right ankle. Values are the mean ± SEM for 4 mice per group (WT cells into C3^−/−^ mice and C3^−/−^ cells into WT mice; *P* < 0.05) or the mean and range for 2 mice per group (C3^−/−^ cells into C3^−/−^ mice and WT cells into WT mice). Very similar results were obtained in a second experiment that included 2 mice per group (C3^−/−^ cells into WT mice and WT cells into C3^−/−^ mice).

The role of leukocyte-derived C3 was assessed in radiation bone marrow chimeras. This experimental approach leads to virtually complete replacement of a mouse's hematopoietic cells with cells from a donor mouse, whereas nonhematopoietic cells remain of host origin. Thus, WT or C3-deficient recipients were lethally irradiated, then reconstituted with unfractionated bone marrow cells from either WT or C3^−/−^ donors (Figure 1A). %Arthritis development after K/BxN serum transfer, whether evaluated by a clinical index or by ankle measurement, correlated perfectly with the ability of the recipients' radioresistant cells to produce C3 (Figure 1B). Furthermore, when 2 mice from each of the critical groups (C3^−/−^ BMCs into WT mice, and WT BMCs into C3^−/−^ mice) continued to receive injections of K/BxN serum for a total of 4 weeks and arthritis was evaluated until day 42, the WT recipients continued to show severe inflammation, while disease never developed in the C3-deficient recipients (results not shown).

**Figure 2:**
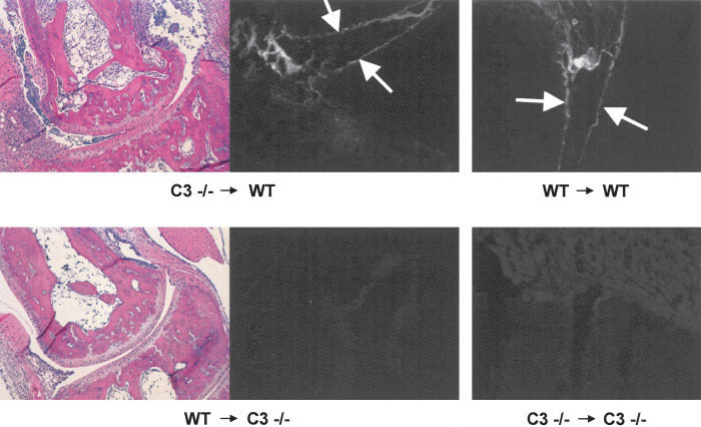
Histopathology of ankle joints, and deposition of C3 in joint tissue. Ankle joints from bone marrow chimeras (as denoted in Figure 1A) that had been injected with K/BxN mouse serum were processed for paraffin sectioning and stained with hematoxylin and eosin (H&E), or were processed for frozen sectioning and stained with fluorescein-labeled antibodies to C3. **Arrows** indicate linear staining on the cartilage surface. Nonlinear staining in the same sections represents deposition in the synovium and subsynovial connective tissue. WT = wild-type. (Original magnification × 100 for H&E-stained sections; × 200 for sections stained with fluorescein-labeled antibodies to C3.)

Histopathologic assessment of ankle joints confirmed the clinical impression of the presence or absence of inflammatory arthritis with the typical features of a dense mononuclear cell infiltrate of the subsynovial connective tissue, neutrophilic joint effusion, loss of cartilage, and marginal erosion of bone (Figure 2). According to immunofluorescence staining, arthritic ankles from WT recipients had prominent deposits of C3 on the cartilage surface and in the synovium, whereas the nonarthritic ankles from C3^−/−^ recipients did not (Figure 2). In short, production of C3 by leukocytes was not required for K/BxN serum–induced arthritis, even with prolonged administration of arthritogenic serum.

### No need for C3 synthesis by joint-resident cells

**Figure 3:**
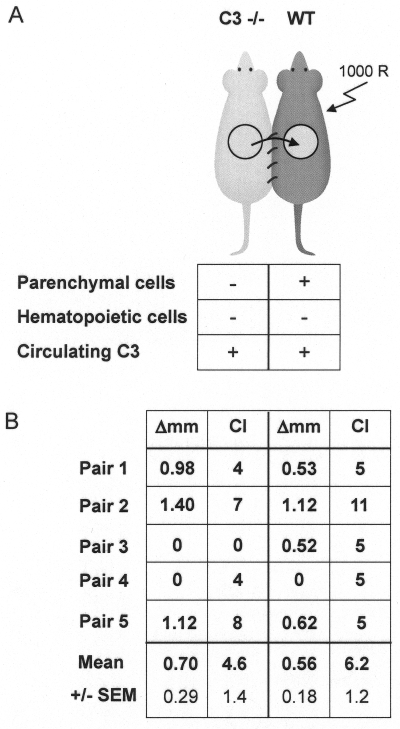
Arthritis susceptibility in parabiotic mice. **A,** Noncirculating parenchymal cells can produce C3 only in the wild-type (WT) partner. C3 cannot be produced by hematopoietic cells derived from either partner: the WT mouse lacks endogenous hematopoietic cells due to the irradiation, while the C3^−/−^ partner is genetically deficient in C3 production by all cell types. Circles represent repopulation of the hematopoietic lineages in the irradiated mouse by hematopoietic cells from the C3^−/−^ partner. C3 synthesized in the WT partner circulates freely through the shared vasculature into the C3-deficient partner. **B,** Joined mice were injected with 0.15 ml K/BxN mouse serum (each partner) twice, 2 days apart, at least 5 weeks after parabiosis surgery. Arthritis severity in each partner is expressed as the maximum increase in thickness of the unconstrained ankle (Δ mm) and as the clinical index (CI) in all 4 paws (maximum severity = 12). Note that the 2 mice in pair 4 had undetectable arthritis in the measured joint but unambiguous arthritis in the other paws, resulting in discrepancies between the change in ankle thickness and the clinical score. Arthritis was somewhat less severe in both partners than is typical for B6 mice, probably due to variability in arthritogenicity of K/BxN serum, because 4 B6 mice treated concomitantly also developed relatively mild disease (maximum CIs = 2, 5, 6, and 9, respectively). In any case, the paw-to-paw variation seen in these experiments (as indicated by discrepancies between the overall CI and the measurement of 1 ankle in several mice) and the absence of arthritis in 1 mouse are typical of what we have seen previously in other settings in which arthritis severity is decreased, such as in relatively resistant mouse strains or with use of smaller-than-usual amounts of serum. R = rads.

**Figure 4:**
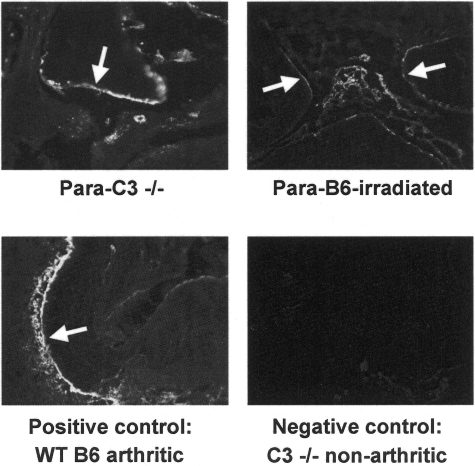
Circulating C3 deposition in arthritic joints. Two weeks after induction of arthritis in parabiotic mice, tissue containing the ankle and midfoot joints was processed for frozen sections, which were then stained with fluorescein-labeled antibodies to C3. Para-B6–irradiated = lethally irradiated wild-type (WT) partner. Para-C3^−/−^ = C3^−/−^ partner (as denoted in Figure 3A). **Arrows** denote staining of cartilage surfaces. Other areas of staining represent synovial and subsynovial deposits. An ankle from a B6 mouse that did not receive K/BxN mouse serum showed no staining (results not shown), similar to the lack of staining observed in the negative control mouse (original magnification × 100).

The next question was whether C3 had to be produced by radioresistant cells locally within the joint, or whether it could be made by parenchymal cells at distant sites and transported to the joints via the circulation. Therefore, we generated mice whose joint-resident cells could not synthesize C3 but whose circulation carried C3 that had been synthesized by cells distant from the joint. C3-deficient mice were surgically joined with WT animals immediately after the latter had been subjected to lethal irradiation in order to ablate circulating blood cells. As illustrated in Figure 3A, the irradiated WT partner in these pairs harbored parenchymal cells that produce C3 and introduce it into the circulation, but lacked hematopoietic cells that might produce C3. The C3^−/−^ partner lacked C3 synthesis by both parenchymal and hematopoietic cells but would be exposed to C3 through its circulation due to anastomosis with the circulation of the WT partner (Figure 3A). Measurement of circulating C3 confirmed equal concentrations of C3 in both partners, at levels ∼25% of those seen in B6 mice (results not shown). Upon transfer of K/BxN serum, clear manifestations of inflammatory arthritis were observed in 4 of the 5 C3^−/−^ partners and all 5 of the irradiated WT partners (Figure 3B). Immunofluorescence staining of ankle joints confirmed that C3 was prominently deposited on the cartilage surface and in the synovium, even in animals whose only source of C3 was through the circulation (Figure 4).

## DISCUSSION

Circulating C3, produced by parenchymal cells distant from the joint, is both necessary and sufficient for induction of disease in the K/BxN mouse model of antibody-mediated arthritis. The serum-transfer system allowed us to focus specifically on the role of C3 in the inflammatory effector phase, after high levels of autoantibodies had been produced. It remains possible that C3 made either by leukocytes or by joint-resident parenchymal cells plays an important role in the initiation phase of autoimmunity, or that these 2 sources make a nonessential contribution to the effector process.

It is reasonable to propose that C3 plays a role in human RA similar to that in the K/BxN serum-transfer system, particularly in the early stages of inflammation or in the reactivation of acute inflammation in previously quiescent joints. Although the specific autoantibody responsible for arthritis in the K/BxN mouse model is observed in only a minority of patients with RA (35), particularly in those with Felty's syndrome or other extraarticular manifestations (36,37), there is now considerable evidence supporting the pathogenicity of autoantibodies in RA. First, the B cell–depleting monoclonal antibody rituximab is highly effective in treating many patients with RA (38). Second, a newly described mouse model links an RA-specific autoantibody (anti–citrulline-containing protein) to murine arthritis (39). The role of complement has not yet been assessed in this context, but findings made previously in other models are likely to remain relevant to RA; that is, the ability of diverse autoantibody responses to generate pathology resembling RA in mice (40) indicates a general sensitivity of joints to antibody-mediated inflammation and supports the generalizability of the principles determined using the K/BxN mouse model and other models.

The hypothesis that local C3 production is important in RA arose from the finding that more C3 is produced in rheumatoid synovium than in osteoarthritic synovium, as determined qualitatively or quantitatively using in situ hybridization to detect messenger RNA (mRNA) for C3 (28,30). Subsequently, several microarray analyses of synovial gene expression in either human patients or rodent models have yielded conflicting data on this point, sometimes showing an increase in synthesis of C3 (41,42) and sometimes not showing such an increase (43–45). Importantly, we also detected mRNA for C3 in mouse synovium by both complementary DNA microarray and quantitative polymerase chain reaction (data not shown), although we did not observe an increase as arthritis unfolded.

The mechanism and kinetics by which circulating C3 gains access to joints are uncertain but not difficult to envision. Because the synovial “membrane” (functionally, a combination of the subsynovial vasculature and the synovial lining) is rather permeable at baseline, roughly in inverse proportion to the mass of the macromolecule (46,47), and because C3, at 185 kd, is not extremely large, it would be expected that an abundant circulating protein of this size would have some passive access to normal synovial fluid. Indeed, although we are not aware of any reports in which C3 has been measured in normal synovial fluid, it has been readily detected in human osteoarthritic joint effusions at concentrations similar to those seen in RA, i.e., ∼0.2–0.5 mg/ml (48,49).

The reason that concentrations in RA are not higher is probably related to local consumption (4–7,48), because inflamed synovium otherwise loses its size-selective filtration and becomes about equally permeable to all macromolecules (46). Even if some increase in synovial fluid C3 is required for it to be locally activated and promote inflammation, increased local vascular permeability may be a very early feature of inflammatory arthritis. For example, studies in the K/BxN mouse serum-transfer model have shown that IgG-containing immune complexes selectively increase the permeability of periarticular vessels to IgG itself (50) and to a higher molecular weight (400 kd) tracer (51). Interestingly, complement is not required for this early vascular permeability (51); therefore, further research on the role of complement in this model will likely focus on downstream events, such as the role of complement receptors, particularly C5aR, on different populations of inflammatory cells.

## AUTHOR CONTRIBUTIONS

Dr. Mathis had full access to all of the data in the study and takes responsibility for the integrity of the data and the accuracy of the data analysis.

**Study design.** Monach, Carroll, Benoist, Mathis.

**Acquisition of data.** Monach, Verschoor, Jacobs, Wagers, Mathis.

**Analysis and interpretation of data.** Monach, Benoist, Mathis.

**Manuscript preparation.** Monach, Benoist, Mathis.

**Statistical analysis.** Monach.
